# T235. SELF ASSESSMENT OF SOCIAL COGNITIVE ABILITY IN SCHIZOPHRENIA: ASSOCIATION WITH SOCIAL COGNITIVE TEST PERFORMANCE, INFORMANT ASSESSMENTS OF SOCIAL COGNITIVE ABILITY, AND EVERYDAY OUTCOMES

**DOI:** 10.1093/schbul/sby016.511

**Published:** 2018-04-01

**Authors:** Philip Harvey, Amy Pinkham, David Penn

**Affiliations:** 1 Leonard M. Miller School of Medicine, University of Miami; 2 University of Texas at Dallas; 3 University of North Carolina

## Abstract

**Background:**

Impairments in self-assessment are commonly found in people with schizophrenia and impairments in introspective accuracy (IA) predict impaired functional outcome. previous studies have suggested mis-estimation of cognitive and functional skills predict impairment in everyday functioning at least as much as ability scores. In this study, we examined self-assessment of social cognitive ability and related these self-assessments to assessments of social cognition from informants, to performance on tests of social cognitive ability, and to everyday outcomes. The difference between self-reported social cognitive abilities and informant ratings was our measure of IA.

**Methods:**

People with schizophrenia (n=135) performed 8 tests of social cognitive abilities. They also rated their social cognitive abilities on the Observable Social Cognition Rating Scale (OSCARs). High contact informants also rated social cognitive ability and everyday outcomes, while unaware of the patients’ other scores. Social competence was also measured with a performance-based assessment and clinical ratings of negative symptoms were also performed.

**Results:**

Patient reports of their social cognitive abilities were uncorrelated with performance on social cognitive tests and with three of the four domains of everyday functional outcomes. IA, in specific overestimation of performance compared to informant ratings, predicted impaired everyday functioning across all four functional domains. IA scores predicted functional outcomes even when the influences of social cognitive performance, social competence, and negative symptoms were considered in regression models. Thus, self-assessment of social cognition had a relatively specific impact social outcomes.

**Discussion:**

Mis-estimation of social cognitive ability was a more important predictor of social and nonsocial outcomes in schizophrenia than performance on social cognitive tests. These results suggest that consideration of IA is critical when attempting to assess causes of everyday disability and when implementing interventions aimed at disability reduction.

